# Treatment of Acute Rheumatism

**Published:** 1853-03

**Authors:** Edwin R. Maxson

**Affiliations:** Adams’ Centre, Jefferson County, N. Y.


ART. V.—*Treatment of Acute Rheumatism.* By EDWIN R. MAXSON, M. D., Adams' Centre, Jefferson County, N. Y.

As acute rheumatism is a disease, the pathology of which has been a matter of doubt, and the treatment consequently various, I here offer a plan of treatment which I have found very satisfactory.

In a severe case, in which the inflammatory fever runs high, if the arms are suffering, I draw from two to four ounces of blood, by cups, from each side of the spine, near the origin of the brachial nerves, between the shoulders. If the lower limbs are suffering, I draw the same quantity, by cups, from each side of the spine, in the lumbar region, near the origin of the crural nerves. This generally relieves the pain in the limbs immediately, and checks the progress of the disease. I procure an evacuation of the bowels by mag. sulph. ℥ss., repeated, if necessary, and then give potassa nit. ℈i. dissolved in a tea-cup full of warm gruel, every three hours, and continue this till the fever and inflammatory symptoms subside, which will generally occur in from four to six days.

At this stage there is generally left slight swelling and some stiffness of the joints. I then discontinue the nitrate and give potassa iodide, gr. x, with vin. colch. gtt. xx, every six hours. This should be continued till the slight swelling and stiffness of the joints subside, which may take place from four to six days. The appetite will, in the mean time, generally become good. The patient then need only be directed to take potassa iodide, gr. v, three times per day, for a few days more, to prevent a relapse and render the cure permanent.

Adams' Centre, N. Y., Jan. 20, 1853.

